# Structural changes of block copolymers with bi-modal orientation under fast cyclical stretching as observed by synchrotron SAXS

**DOI:** 10.1039/c5sm00360a

**Published:** 2015-03-17

**Authors:** J. Stasiak, J. Brubert, M. Serrani, A. Talhat, F. De Gaetano, M. L. Costantino, G. D. Moggridge

**Affiliations:** a University of Cambridge , Department of Chemical Engineering and Biotechnology , Pembroke Street , Cambridge , CB2 3RA , UK . Email: js744@cam.ac.uk; b Politecnico di Milano , Department of Chemistry , Materials and Chemical Engineering , Piazza L. da Vinci 32 , 20133 Milan , Italy

## Abstract

Load-bearing tissues are composite materials that depend strongly on anisotropic fibre arrangement to maximise performance. One such tissue is the heart valve, with orthogonally arranged fibrosa and ventricularis layers. Their function is to maintain mechanical stress while being resilient. It is postulated that while one layer bears the applied stress, the orthogonal layer helps to regenerate the microstructure when the load is released. The present paper describes changes in the microstructure of a block copolymer with cylindrical morphology, having a bio-inspired microstructure of anisotropic orthogonally oriented layers, under uniaxial strain. To allow structural observations during fast deformation, equivalent to the real heart valve operation, we used a synchrotron X-ray source and recorded 2D SAXS patterns in only 1 ms per frame. The deformation behaviour of the composite microstructure has been reported for two arrangements of the cylinders in skin and core layers. The behaviour is very different to that observed either for uniaxially oriented or isotropic samples. Deformation is far from being affine. Cylinders aligned in the direction of stretch show fragmentation, but complete recovery of the spacing between cylinders on removal of the load. Those oriented perpendicular to the direction of stretch incline at an angle of approximately 25° to their original direction during load.

## Abstract 2

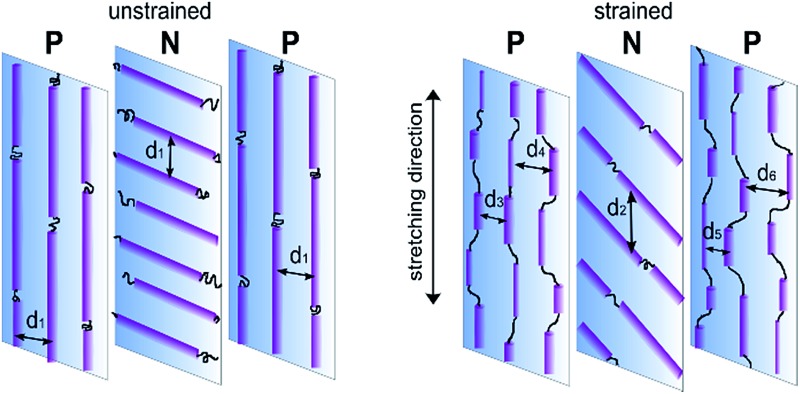
Here we examine a block copolymer with cylindrical morphology having a bio-inspired microstructure of anisotropic orthogonally oriented layers and report changes of the microstructure under uniaxial strain.

## Introduction

Structure property relationships and the deformation mechanisms of triblock copolymers based on thermoplastic elastomers containing glassy and rubbery segments have been intensively investigated to explore suitable industrial applications.^1–7^ It has been shown that the deformation mechanisms of block copolymers differ from those of homopolymers and strongly depend on the morphological pattern and microdomain orientation^8–16^ as well as deformation rate.^17–19^ Thus, examination of the materials should be performed using realistic conditions for a specific application.

It is well known that this group of block copolymers can align their microstructure during processing. It is common to obtain unidirectionally aligned materials by extrusion, drawing or compression.^20–25^ Furthermore we have recently demonstrated that block copolymers, like poly(styrene-*block*-isoprene-*block*-styrene) with cylindrical morphology, can form a layered bi-directional microstructure resulting from shear flow and elongational flow regimes present during slow injection moulding.^26^ Such a microstructure is similar to many fibre containing biological tissues, for example heart valve leaflets.^27–29^ The microstructure results in anisotropic mechanical properties mimicking those of the native tissue, and having potential for application as a material for prosthetic polymeric heart valve leaflets.

In real conditions of heart valve functioning a blood pressure acts on the leaflets causing the valve to open or close. For the aortic valve, normal blood pressure drop is 120 mm Hg but may reach 180 mm Hg for hypertension conditions. This load is released during the systole period and the cycle repeats 60 to 100 times per minute at the normal resting rate, depending on the person and physical condition. Higher heart rate is usually associated with intense exercising or some medical conditions like arrhythmia or tachycardia. To evaluate suitable materials for prosthetic heart valve leaflets, they need to be subjected to deformation at a rate corresponding to real conditions. Our earlier paper reported measurements of microstructure during cyclical deformation equivalent to normal heart beat rate, 68 bpm (beats per minute) and unidirectionally oriented materials. During cyclical deformation, SAXS patterns were recorded for 10 ms and the microstructure followed over 10 000 cycles.^30^ Microstructural observations using synchrotron small angle X-ray scattering (SAXS) provided information at the microscopic level about the polymer packing and cylinders orientation. As with this previous study, the present paper shares the aim of providing insight into microstructural changes during fast deformation to understand features affecting the material's mechanical performance. Here however, we pushed the limit of SAXS time resolution to only 1 ms and report the changes of microstructure over 20 000 cycles. Such high time resolution allowed investigations of higher frequency cyclical deformation corresponding to a heart rate of 120 bpm. Moreover, this study is focused on measurements of the bi-directionally oriented microstructure, obtained by slow injection moulding of cylindrical block copolymers. The structure contains skin and core layers oriented perpendicularly to each other. Reporting the evolution of such complex microstructure during fast mechanical deformation is of general scientific interest as well as relevant for the proposed applications of these materials.

## Experimental

Cylinder forming, poly(styrene-*block*-isoprene-*block*-styrene), block copolymer was examined, manufactured by Kraton Polymers, commercial product name Kraton D1164P. It is a linear block copolymer having: 30% wt styrene fraction, molecular weight *M*_w_ = 131 kg mol^–1^ and polydispersity index *M*_w_/*M*_n_ = 1.1, where *M*_n_ is the number average molecular weight. The linear A–B–A triblock copolymers of styrene and isoprene is investigated as its simple structure makes it a model copolymer, which can serve as a basis for understanding more complicated systems.

Disc shape samples of 0.25 mm thickness were prepared by injection moulding of the block copolymer melt at 160 °C and 2 × 10^–8^ m^3^ s^–1^ injection rate. The slow rate injection, into the centre of the disc, induced a bimodal microstructure orientation of the styrenic cylinders. The composite morphology features two surface layers with cylinders aligned in the flow direction, and a central core layer, where the cylinders are aligned transverse to the flow direction. In other words the outer-skin layers have cylinders oriented radially from the centre of the disc, whilst the core layer contains cylinders oriented circumferentially around the centre of the disc. More details about the bi-modal microstructure can be found elsewhere.^26^

**Fig. 1:**
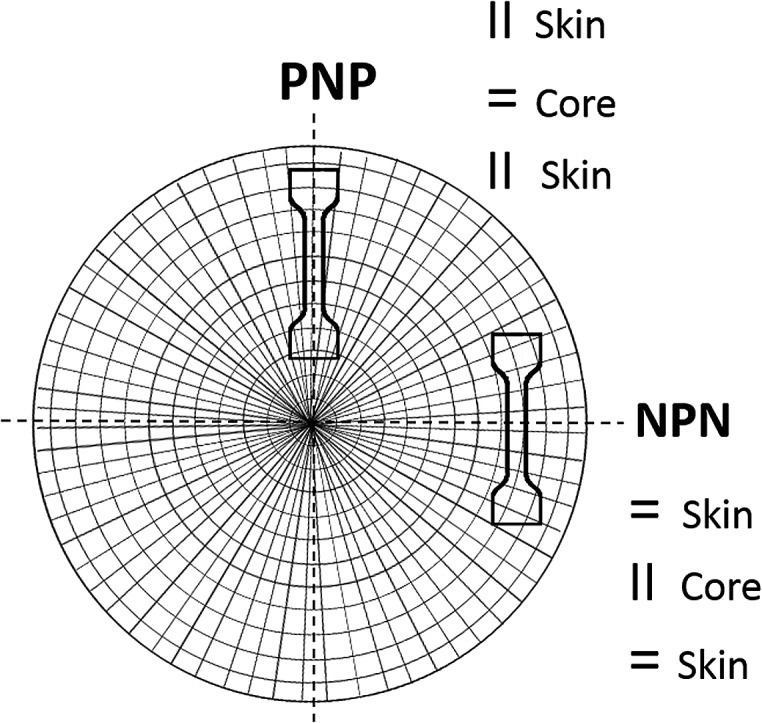
A schematic of the specimens layout with reference to the disc's radius resulting in microstructures having various orientation of the cylinders within skin and core layers for sample PNP and NPN.

Bone shaped specimens were cut out for stretching from the disc sample at 0° (PNP sample) and 90° (NPN sample) with respect to sample radius, as shown in Fig. 1. This layout of the specimens allowed investigation of two configurations of cylinder orientation within the core and skin layers with respect to the stretching direction. Sample PNP displayed normal orientation (N) within the core, sandwiched between skin layers oriented parallel (P), while sample NPN exhibited parallel (P) orientation within the core and normal (N) within the adjacent skin layers.

SAXS experiments were performed on the I22 beamline at the Diamond Light Source synchrotron at Harwell, UK. The beamline was tuned to operate at a beam energy of 12.4 keV, giving a wavelength of 1 Å, and the camera length was 6 m. Each X-ray frame was collected in only 1 ms. This was possible by using the RAPID detector system on I22, which offered unique capabilities for the experiment, such as short time frames at very high rates, therefore eliminating stroboscopic effects. More information about the beamline and detector can be found elsewhere.^31^ In order to follow structural changes in the polymer during deformation using a scattering measurement, the incident beam was maintained at the same position on the sample throughout the deformation process.

The load stress–strain curves for the samples were evaluated using a custom made instrument (stretching device) equipped with a data acquisition system. Two stepper motors moved retaining clamps in opposite directions applying symmetrical uniaxial strain to the sample. Stress as a function of strain was recorded simultaneously while SAXS data was collected. Reduced physical dimensions and weight made the instrument portable and easily installed and utilised on the synchrotron beamline. Samples were clamped and stretched vertically at a rate of 300 mm s^–1^. The size of the specimens after clamping was 30 mm length, 7 mm width and 0.25 mm thickness.

**Fig. 2:**
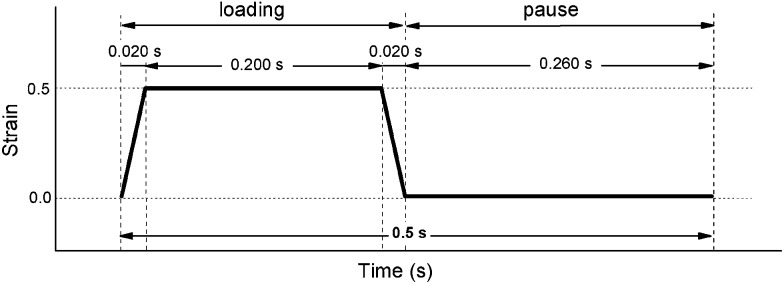
A schematic of the experimental stretching cycle.

The stretching cycle, schematically shown in Fig. 2, imitates the conditions of blood pressure drop in aortic valve, where loading corresponds to the backpressure acting on the closed valve during diastole. Our computational stress analysis of a heart valve leaflet made from the block copolymer, indicate that the maximum stress is generated in commissures, the region where the free edge of the leaflet meets the aorta, and achieves about 1 MPa at 120 mm Hg backpressure. To simulate such conditions the samples were subjected to an equivalent uniaxial strain *ε* = 0.5, where *ε* = Δ*L*/*L*_0_, Δ*L* is the increase of sample length and *L*_0_ is the initial sample length. Thus the experiment represents a situation within the most stressed part of the leaflet.

The duration of a single cycle is here only 0.5 s to achieve a frequency equivalent to a heart rate of 120 bpm.

Simultaneous tension and SAXS data were collected after sample preconditioning of 1000 stretching cycles.

## Results and discussion

### Effect of long-term cyclical deformation

**Fig. 3:**
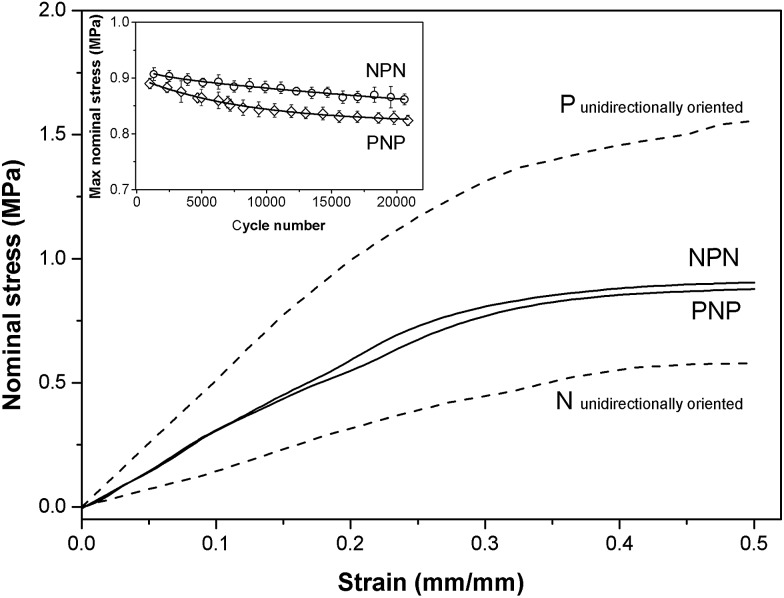
Stress–strain data for samples PNP and NPN and unidirectionally oriented samples stretched in direction parallel to cylinders (P) and normal to cylinders (N). Inset graph presents maximum nominal stress as a function of cycle number.

The stress–strain relationship for samples PNP and NPN having layered cross-wisely arranged microstructures is shown in Fig. 3. As the samples contain layers oriented parallel and perpendicular to the stretching direction, the stress–strain curves lie between those for unidirectionally oriented (prepared by compression moulding) samples stretched in directions parallel (P) and normal (N) to the cylinders' longitudinal axis. PNP and NPN samples showed similar stress–strain trace behaviour, indicating that the fraction of the parallel and perpendicular orientation is approximately equal in both samples. The slight discrepancy at higher strain suggests a small predominance of the circumferentially oriented layer located in the middle of the sample, which for the NPN configuration is stretched parallel to the cylinders, generating greater stress. Over 20 000 stretching cycles both samples displayed slight stress softening, observed as a drop of the maximum nominal stress, presented in the inset of Fig. 3.

**Fig. 4:**
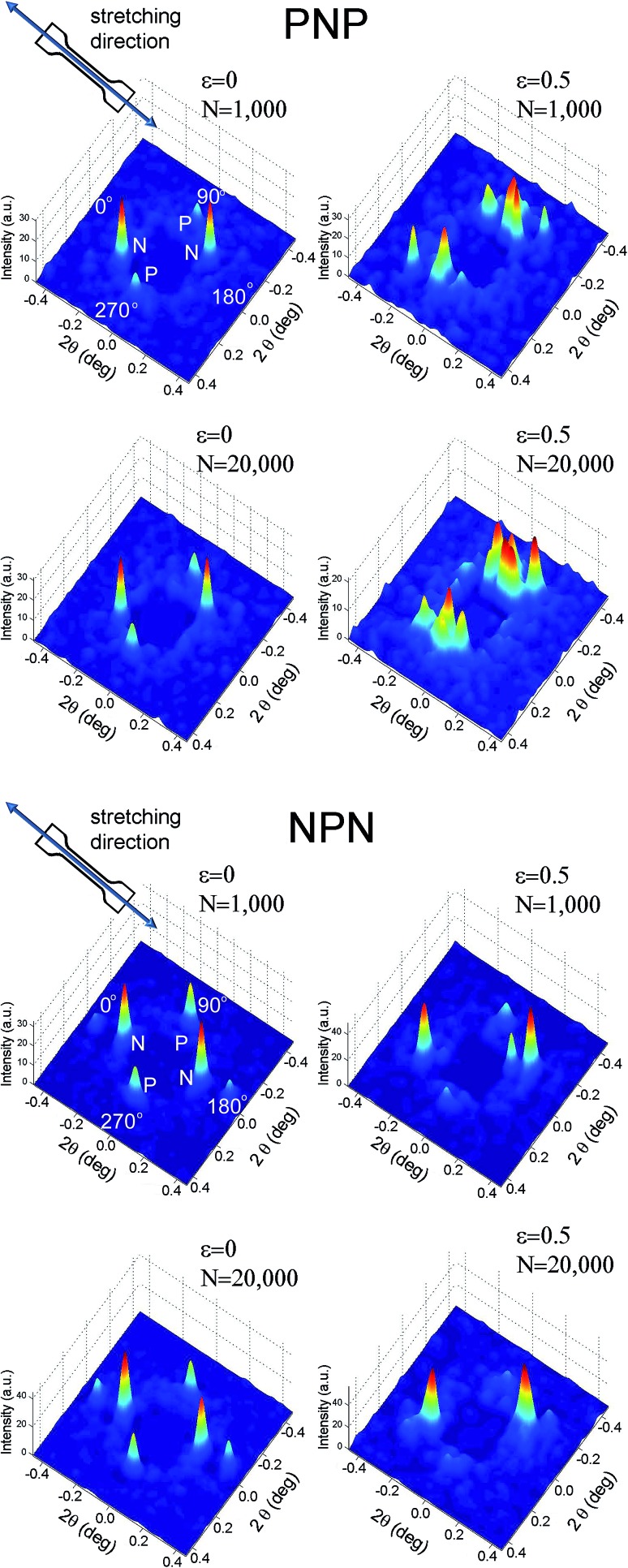
Surface plots of X-ray intensity for PNP and NPN samples collected over 1 ms for strained and unstrained samples and after 1000 and 20 000 cycles.

SAXS images, collected over 1 ms, for PNP and NPN samples are shown as surface plots in Fig. 4, where peaks of intensity represent the scattering reflections. The figure contains SAXS images of the relaxed microstructure (*ε* = 0) and at the maximum strain (*ε* = 0.5) at the beginning of experiment, after about *N* = 1000 cycles and in the end of the experiment, after about *N* = 20 000 cycles.

Four scattering maxima can be recognized, a pair at azimuthal angles 0° and 180°, and another at 90° and 270°. Such patterns demonstrate a layered structure, where the cylinders are oriented vertically in one layer and horizontally in another. In fact, as we described in a previous paper,^26^ the structure comprises of two skin layers oriented along the flow direction and a core layer oriented orthogonally. By integration of the area of the first order peaks, the fraction of core and skin layers within the irradiated sample volume was evaluated. For PNP samples the skin layers P occupied 34% of the total sample volume covered by the incident X-ray beam and the core layer N – the remaining 64%. NPN sample contained 68% of the skin layers N and 32% of the core layer P. This is consistent with local variations of skin and core layers' thicknesses between 30 and 70% across each sample as determined by X-ray mapping. The macroscale sample morphology, characterised by the mechanical response (presented in Fig. 3), which averages all the local structural features, comprises of roughly a half of the structure oriented parallel and a half oriented perpendicular to stretching direction.

Deformation of the local microstructure has been studied by monitoring changes in X-ray patterns upon load: sector integration of the reflections corresponding to skin and core layer alignment was carried out separately. Often, the interpretation of SAXS data is limited to the estimation of average spacings between microdomains, from the observed peak position, by a simple application of the Bragg's equation. The morphological changes resulting from deformation of block copolymers are very complex, and many structural parameters, such as sizes of domains, their orientation distribution, interdomain distances and their relative uniformity can change simultaneously. Here we also investigate the shape and intensity of the X-ray reflections to understand development of the microstructure upon load and to allow correlation with the macroscopic deformation.

**Fig. 5:**
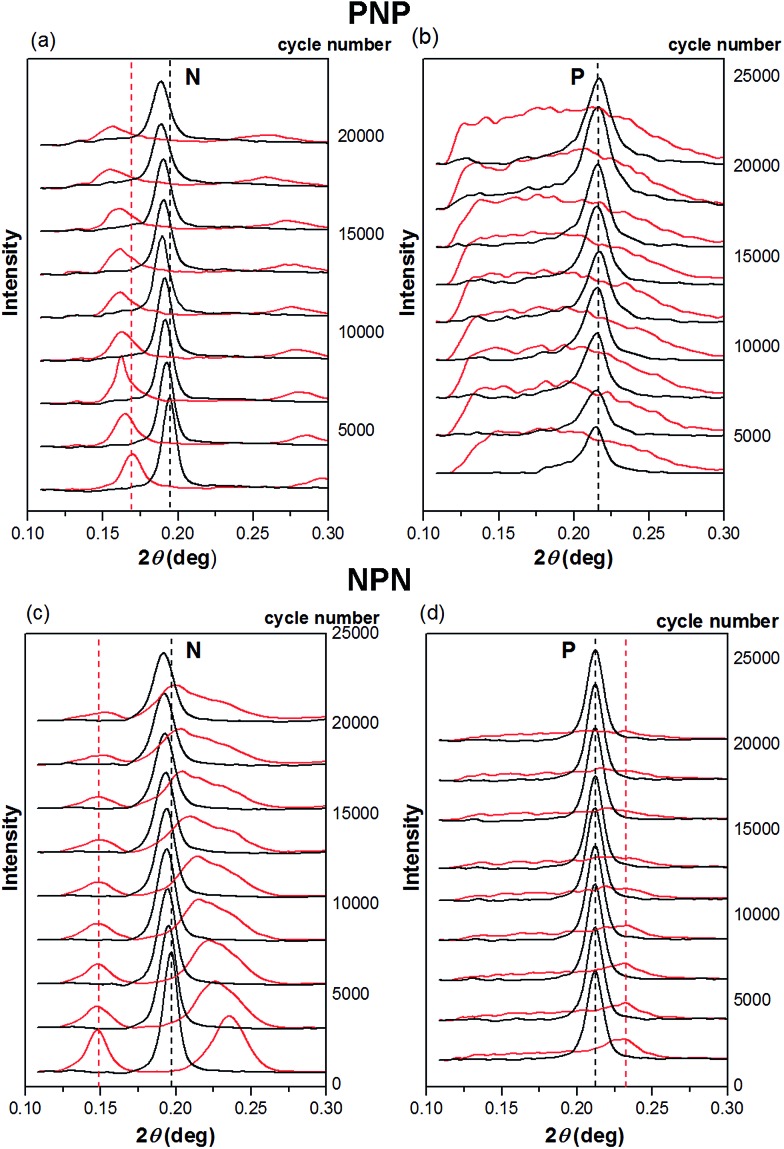
SAXS profiles' change with cycle number for PNP sample within (a) core layer N (b) skin layers P and NPN samples within (c) skin layers N and (d) core layer P. Black lines represent relaxed microstructure, red lines stretched up to *ε* = 0.5. Dashed lines represent positions of first order reflection at the beginning of the experiment.

**Fig. 6:**
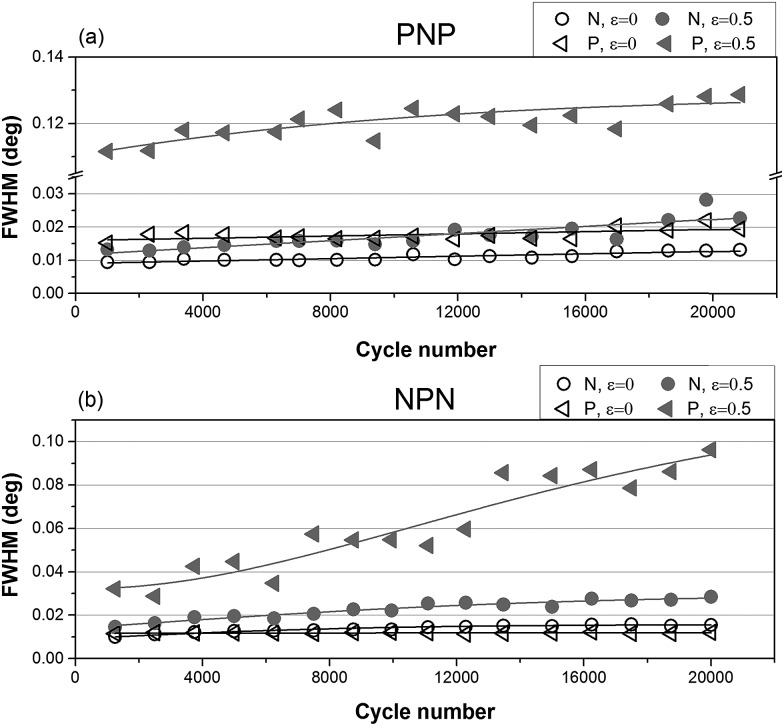
FWHM as a function of cycle number for (a) PNP sample and (b) NPN sample.

**Fig. 7:**
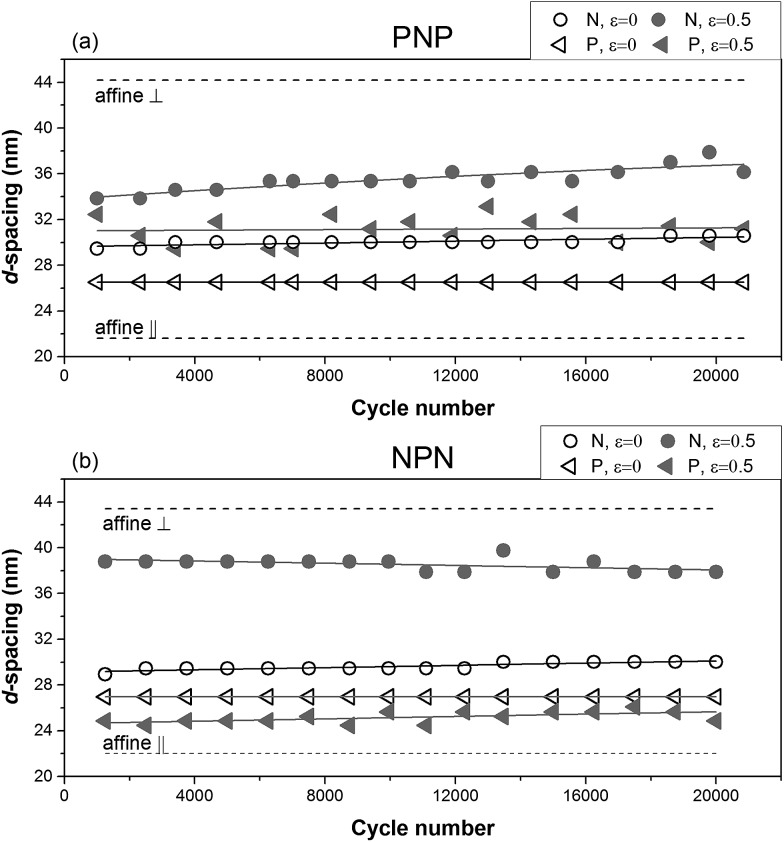
Interdomain spacing as a function of cycle number for (a) PNP sample and (b) NPN sample.

Despite the low counts observed, 1 ms frames (Fig. 4) show noticeable differences between intact and strained microstructures. Apart from the expected shift in 2*θ* angle position, the peaks corresponding to the parallel orientation became broad and poorly resolved, whereas an azimuthal shift was observed for the reflections representing alignment normal to the strain. Structural changes caused by longer term stretching up to 20 000 cycles are less distinct and further processing of the SAXS data is necessary to spot them. Hence, for Fig. 5–7, one hundred 1 ms SAXS frames were summed, at relaxed and strained conditions, to amplify the measurements and reduce data noise. Fig. 5 illustrates selected SAXS profiles for relaxed and strained samples as a function of increasing number of stretching cycles. For both PNP and NPN samples, a shift in location of the first order peak occurred in layers N, oriented normal to the strain. The micro-scale reversibility of the deformation weakened with cycle number (Fig. 5a and c). The shift in position of the primary scattering reflections towards smaller 2*θ* angle for relaxed sample, indicates that the microstructure stretches less and less as the cycle number increases. This can be attributed to residual elongation of the sample on the macroscopic scale and bending when the load was released.

On the contrary, the layers P oriented parallel to strain direction, showed excellent reversibility of the microstructure to the original unstrained arrangement (Fig. 5b and d). Strained samples however, displayed a significant broadening of the primary reflection. In some cases, as in Fig. 5b, the shape of the distribution curve became almost flat, making the peak maximum and so the spacing between domains, difficult to identify. As the hard microdomains are oriented parallel to the applied strain in layers P, they may be fragmented by stress transfer from the surrounding elastic matrix. It is supposed that broken cylinders of various lengths distribute in the direction of strain. Occasionally cylinders originally located in neighbouring lattices may be pulled apart, simultaneously bringing closer another cylinders originally associated to a secondary lattice.

This results in a distribution of the interdomain spacing between parallel cylinders, with some segments showing the expected decrease of the spacing caused by lateral compression of the sample during uniaxial stretching, and others showing an increase of the horizontal spacing in strained sample. The resulting SAXS profile sums up all the morphological features along the sample depth penetrated by the X-ray beam. The broad distribution is then explained by variations of interdomain spacings within P layers. Surprisingly all these slips and shifts in cylinders locations are almost perfectly reversible when the external stress is removed, allowing for reformation of the original structure. Pakula *et al.* ^32^ have reported a similar mechanism for uniaxial deformation causing fragmentation of the domains. In addition to these authors' work, our studies show that for block copolymers with higher styrene fraction (30% wt), significant fragmentation occurs at lower strain compared to lower styrene fraction materials (18%), which we have reported previously.^30,33^

While positions of the peak depend on the distance between cylinders, shapes of the integrated intensity profile depend on the orientation of particles with respect to the lattice as well as domain size and lattice distortion in the material. The influence of long-term cyclical deformation on shape of the primary X-ray scattering reflections has been investigated by means of Full Width of Half Maximum (FWHM) analysis.

Fig. 6 shows that peak broadening with cycle number is marginal for relaxed samples and almost absent for P layers at zero strain. Broadening is noticeable for stretched layers N and becomes substantial for P layers at *ε* = 0.5. Moreover, for sample PNP significant broadening had already taken place early in the long-term experiment, while NPN sample showed a more gradual increase of FWHM with cycle number. This can be associated with the different location of the parallel layer(s) in samples PNP and NPN. It is known that the parallel orientation generates greater stress upon load compared to perpendicular orientation.^34,35^ It is postulated that the surface layer may respond more sensitively to the applied stress, because there is only one neighbouring N layer, where the increase of stress can be dissipated. By contrast, for the NPN sample, where the critical parallel layer is located in the middle, fragmentation is less sudden and peak broadening continuously increased throughout the experiment.

**Fig. 8:**
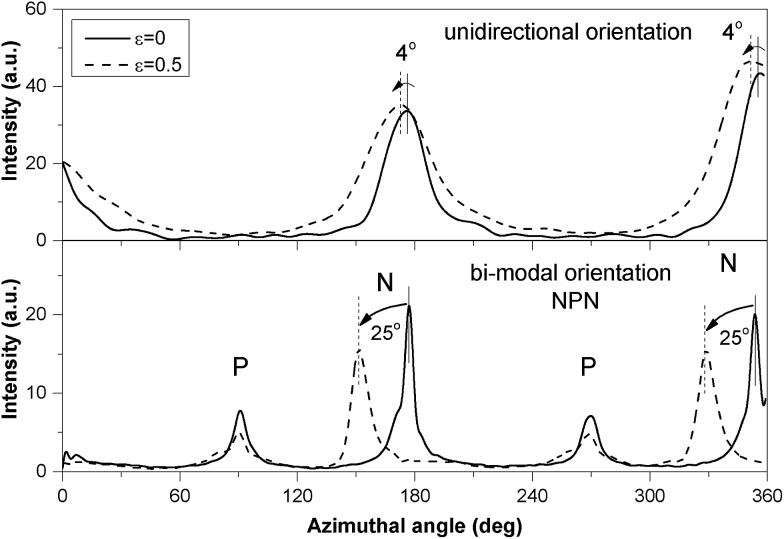
Azimuthal intensity profiles for sample NPN and unidirectionally oriented sample N at *ε* = 0 and *ε* = 0.5.

The observed changes in interdomain spacing over the 20 000 stretching cycles are presented in Fig. 7. It is clear that the periodicity of the scattering bodies in both strained samples did not conform to affine deformation. This means that neither the longitudinal increase of interdomain distance in layers N, nor the lateral decrease of *d*-spacing in layers P correspond to the macroscopic change of the samples' length. For P layers, we have identified structure fragmentation into smaller regions being responsible for local changes in deformation behaviour, and this may be responsible for considerable deviation from affine deformation. For N layers, however, a potential morphological explanation for the apparent strain deficit is an inclination of the perpendicular cylinders towards the direction of strain. 25 degrees azimuthal shift of the peak corresponding to the N layer in strained NPN sample can be seen in Fig. 8. For comparison the uniaxially oriented sample, moulded by compression and stretched normal to the cylinders' orientation showed only 3–4° inclination at the same strain.

In view of the changes in peaks broadening, no significant fragmentation of the perpendicular cylinders occurs within the tested strain range. Instead, the deformation mechanism involved elongation of the surrounding matrix causing both movement of the microdomains relative to each other and reorientation so that the long axes of the cylinders rotated towards the direction of applied strain.

**Fig. 9:**
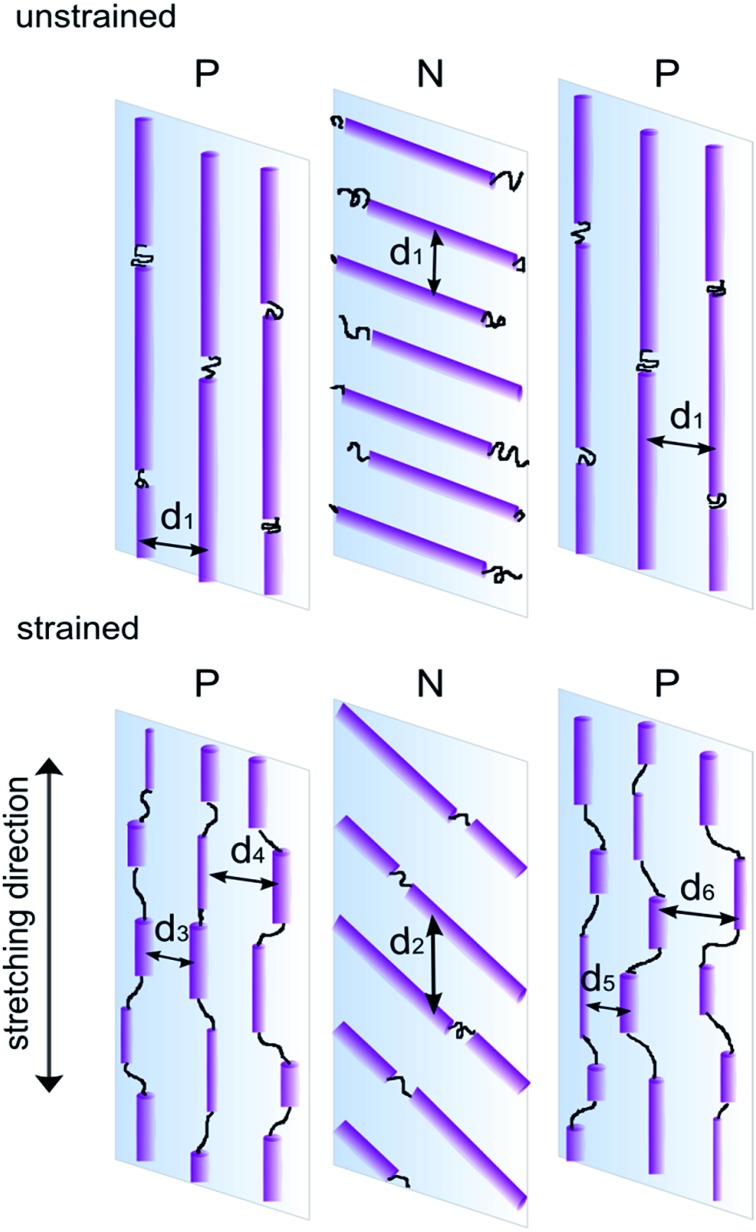
Illustration of proposed deformation mechanism for microstructure comprising orthogonally oriented skin and core layers.

Based on the above analysis a model of deformation within the skin-core layered structure has been proposed and is schematically presented in Fig. 9. The two skin layers and orthogonally aligned core layer, bounded together in a single material, show two different deformation mechanisms. Microdomains oriented parallel to the applied strain may be fragmented by stress transfer from the surrounding matrix. The resulting scattering bodies are shorter, but not separated affinely, as would be expected on the basis of the overall change of sample length. The initial periodicity *d*_1_ transforms into a range of domain spacings illustrated schematically as *d*_3_, *d*_4_, *d*_5_, *d*_6_. In practice, elongation and fragmentation of the domains are likely to occur simultaneously, so changes in the average morphological parameters obtained from the SAXS data will reflect a balance between the two processes.

Segments aligned perpendicular to the strain are pulled apart by elongation of the elastic matrix. Here rotation of the cylinders towards the direction of stretching predominates, with no significant fragmentation of the domain.

For both samples, with different structural arrangements within the core and skin layers, the deformation and the resulting estimates for FWHM and *d*-spacing behaved essentially the same, with microdomains becoming rotated in the strain direction for layers N, and fragmentation occurring in the P layers. However the external position of P layers in the PNP sample made them more prone to fragmentation than in case of the NPN sample, where the parallel layer is located in the middle.

### Dynamic microstructural changes

We have previously demonstrated that changes of maximum intensity of the first order reflection as well as the total intensity of the X-ray pattern can be correlated with a load-extention curve and make excellent markers to monitor the dynamic structural deformation in this materials.^30^ Following the dynamic intensity markers is especially convenient in measurements involving acquisition of a large number of X-ray patterns. This study shows that these markers are also suitable for the very short 1 ms measurements of the complex microstructure.

**Fig. 10:**
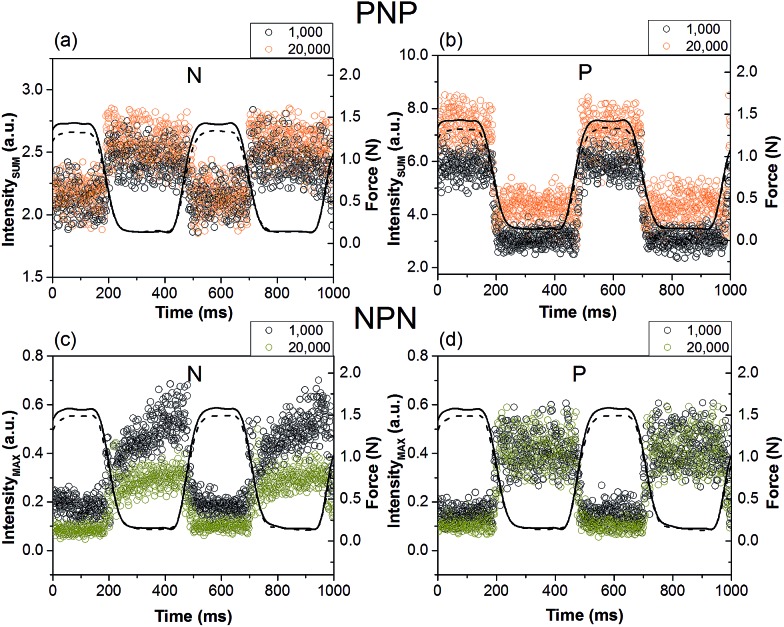
Dynamic intensity markers as a function of applied force after 1000 and 20 000 cycles: (a) and (b) total integrated intensity for PNP sample and layers oriented normal N and parallel P to stretching direction; (c) and (d) maximum intensity of the first order reflection for NPN sample and layer N and P, respectively. Force at 1000 cycles is represented by solid line and at 20 000 cycles by dashed line. Each data point corresponds to an individual X-ray frame collected for 1 ms.

Fig. 10a and b shows real-time changes of the total integrated intensity of the X-ray patterns for sample PNP, where each data point corresponds to a single X-ray frame collected over 1 ms. Due to the low number of counts in these short frames, some variation of the intensity between frames was observed. However, the change between the non-deformed and deformed microstructure is clearly evident. The total integrated intensity correlates well with applied force. For both samples, the intensity over two deformation cycles is shown separately for skin and core layers. We observed either increase or decrease of the intensity in the stretched state, depending on the X-ray pattern seen for each layer respectively. This in turn was influenced by packing of the cylinders, degree of orientation and domain size. For example a substantial increase of the total pattern intensity was observed at the skin layer P (Fig. 10b), where significant fragmentation occurred as a result of stretching and the primary scattering peak broadened in 2*θ* angle. Conversely, the core N layer showed a small decrease of total intensity in the strained state (Fig. 10a), most likely due to a reduction of sample thickness and phase contrast.

An important process that affects deformation of a polymer is relaxation. This process starts when the load is taken off the sample or when the sample is loaded. The resulting microstructure can be greatly affected in terms of residual strain and packing of the cylinders. The changes of maximum intensity of the first order reflection with applied force for sample NPN are shown in Fig. 10c and d. It is evident for layer N that the maximum intensity of the first order reflexion changes with time during the pause period lasting 260 ms. After 20 000 cycles the microstructure relaxed less than at the beginning of the experiment and a smaller slope of the intensity was observed. It is then possible that rearrangement of the microstructure was not fully accomplished within the short pause period, which is in agreement with the measured spacing between the cylinders at the beginning and in the end of the experiment (see Fig. 7b). After 1000 cycles, the distance between cylinders changed from 28.9 nm in the relaxed state to 38.8 nm in the stretched state, and after 20 000 cycles the range shrank from 30.0 nm to 37.9 nm.

No significant relaxation was observed for N layer in the core of sample PNP nor in either of the P layers, which may suggest that the reversibility is instantaneous and completed within the 260 ms.

**Fig. 11:**
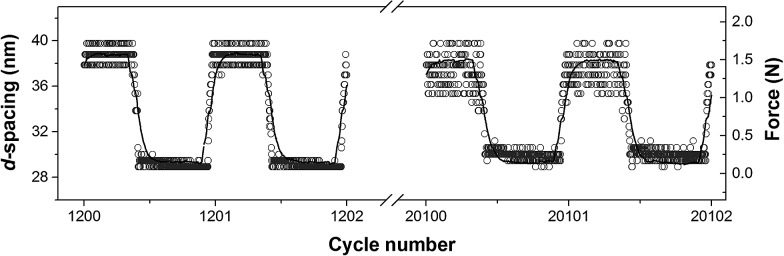
Dynamic changes of *d*-spacing (circle marker) with force (solid line) and cycle number for perpendicular layer N of the NPN sample. Each data point corresponds to an individual 1 ms SAXS frame.

Finally, the dynamics of the *d*-spacing change during deformation can also be correlated with the applied force as seen in Fig. 11. Due to the large number of the short time SAXS frames collected, observation of the transitional region between the relaxed and strained microstructure is possible. For the N layer of the NPN sample, shown in Fig. 11, the domain spacing clearly follows closely the applied stress–time curve. Due to the longer-term structural changes, discussed previously, an increase of variation between *d*-spacings was observed with increasing number of deformation cycles.

## Conclusions

Load-bearing tissues are composite materials that depend strongly on anisotropic fibre arrangement to maximise performance. One such tissue is the heart valve, with orthogonally arranged fibrosa and ventricularis layers. Their function is to maintain mechanical stress while being resilient. It is postulated that while one layer bears the applied stress, the orthogonal layer helps to regenerate the microstructure when the load is released.

The present paper describes changes in the microstructure of a block copolymer with cylindrical morphology, having a bio-inspired microstructure of anisotropic orthogonally oriented layers, under uniaxial strain. The deformation behaviour of the composite microstructure has been reported for two arrangements of the cylinders in skin and core layers. It was found that the parallel layer is able to recover the non-deformed lattice periodicity immediately after the load is released. Although the deformation is reversible, it is not affine. In view of the changes in peak broadening, microdomain fragmentation is likely to be occuring for the parallel layers. Since the resulting fragments can be expected to bisect the distances between the original scattering bodies, this process can explain the deviation from the expected lateral distance.

The perpendicular layers also do not show affine deformation. The apparent strain deficit in the longitudinal direction is explained by inclination of the cylinders in direction of the applied strain. The rotation is much greater in the bi-modal structure described here, than for unidirectionally oriented material at the same strain.

The reversibility of the bi-modal microstructure is a complex function of the orientation, stretching rate and relaxation time. Even though we observed various changes of the microstructure for parallel and perpendicular layer, reversibility of the entire microstructure was still possible over the 20 000 fast stretching cycles. Longer-term dynamic experiment is necessary to clarify the number of cycles up to a critical structural defect.

1 ms time-resolved SAXS frames contain fundamental structural information and can be used to follow structural changes during fast deformation. Total integrated intensity of such short frame and maximum intensity of the first order reflection change with applied force and are convenient markers for dynamic observation of the microstructure.

Although results of deformation of poly(styrene-*block*-isoprene-*block*-styrene) have been presented here, qualitatively similar behaviour was also observed for other microphase separated materials, like poly(styrene-*block*-isoprene/butadiene-*block*-styrene) with 19% wt styrene and poly(styrene-*block*-isobutylene-*block*-styrene) having 30% wt styrene.
